# The Impact of Exercise on Cardiotoxicity in Pediatric and Adolescent Cancer Survivors: A Scoping Review

**DOI:** 10.3390/curroncol29090500

**Published:** 2022-09-03

**Authors:** Stephanie J. Kendall, Jodi E. Langley, Mohsen Aghdam, Bruce N. Crooks, Nicholas Giacomantonio, Stefan Heinze-Milne, Will J. Johnston, Melanie R. Keats, Sharon L. Mulvagh, Scott A. Grandy

**Affiliations:** 1 School of Health and Human Performance, Dalhousie University, Halifax, NS B3H4R2, Canada; 2 Beatrice Hunter Cancer Research Institute, Halifax, NS B3H4R2, Canada; 3 Faculty of Health, Dalhousie University, Halifax, NS B3H4R2, Canada; 4 Department of Pediatrics, IWK Health, Halifax, NS B3K6R8, Canada; 5 Department of Medicine, Division of Cardiology, Nova Scotia Health, Halifax, NS B3H3A7, Canada; 6 Department of Pharmacology, Dalhousie University, Halifax, NS B3H4R2, Canada; 7 Department of Medicine, Division of Medical Oncology, Nova Scotia Health, Halifax, NS B3H4R2, Canada

**Keywords:** exercise, cardiotoxicity, cancer, cancer survivor, pediatric, adolescent

## Abstract

Childhood and adolescent cancer survivors are disproportionately more likely to develop cardiovascular diseases from the late effects of cardiotoxic therapies (e.g., anthracycline-based chemotherapy and chest-directed radiotherapy). Currently, dexrazoxane is the only approved drug for preventing cancer treatment-related cardiac damage. While animal models highlight the beneficial effects of exercise cancer treatment-related cardiac dysfunction, few clinical studies have been conducted. Thus, the objective of this scoping review was to explore the designs and impact of exercise-based interventions for managing cancer treatment-related cardiac dysfunction in childhood and adolescent cancer survivors. Reviewers used Joanna Briggs Institute’s methodology to identify relevant literature. Then, 4616 studies were screened, and three reviewers extracted relevant data from six reports. Reviewers found that exercise interventions to prevent cancer treatment-related cardiac dysfunction in childhood and adolescent cancer survivors vary regarding frequency, intensity, time, and type of exercise intervention. Further, the review suggests that exercise promotes positive effects on managing cancer treatment-related cardiac dysfunction across numerous indices of heart health. However, the few clinical studies employing exercise interventions for childhood and adolescent cancer survivors highlight the necessity for more research in this area.

## 1. Introduction

Antineoplastics are increasingly effective at treating malignancies and minimizing damage to healthy tissues [1]. However, many standard cancer treatments exhibit cardiotoxic effects leading to the development of late or acute cardiovascular complications. Chest-directed radiotherapy and anthracycline treatments are of particular concern as they can cause severe cardiac complications [2,3,4,5,6,7,8]. The risk of cancer treatment-related cardiac dysfunction (CTRCD) is related to the dose of cardiotoxic treatment received, and specific populations are at a higher risk.

Childhood and adolescent cancer survivors (CACS) have a remarkably elevated risk of developing CTRCD. The incidence of CTRCD is widely reported in CACS [2,7,8] such that they are two times more likely to develop cardiac abnormalities and have an 11-fold higher cardiovascular disease-related mortality risk than their healthy siblings [9]. Cardiac-related diseases are also the leading cause of non-malignant deaths in CACS [4], likely because CACS often live long into remission, allowing the latent effects of their cancer treatments to manifest. CACS commonly develop left ventricular (LV) systolic dysfunction, heart failure, myocarditis, pericarditis and arrhythmias [2,3,4,5,6,7,8]. Such effects can develop acutely, but often do not develop until many years into survivorship [2]. Thus, the high incidence and severity of CTRCD in CACS emphasizes the need to mitigate and manage cases.

Currently, CTRCD prevention methods focus on minimizing radiation exposure and cumulative anthracycline dose. Strategies also include liposomal encapsulated anthracyclines delivery [10] and improvements in radiation therapy that exhibit reduced cardiotoxic properties while maintaining high antineoplastic effects [11]. Unfortunately, these strategies do not apply to all cases [11] and do not fully mitigate CTRCD risk [10]. Dexrazoxane is the only widely used pharmaceutical for preventing anthracycline-induced CTRCD [3,12]. Unfortunately, dexrazoxane’s effectiveness is not established in preventing radiation-induced damage as its effects have only been explored in animal models [13], it must be administered concurrently with anthracycline treatment [14], and it may exhibit chronic side effects such as decreased fertility [14]. Other pharmaceuticals, such as conventional heart medications like beta-blockers, statins, and renin-angiotensin-aldosterone system blockades, can be administered upon detecting asymptomatic LV dysfunction to prevent further development of symptomatic heart failure disease [15]. However, conventional heart medications are often not administered timely as routine cardiac imaging is uncommon in CACS and frequently fails to detect asymptomatic LV dysfunction. Consequently, conventional heart medications are often not prescribed until symptomatic, irreversible dysfunction occurs. The severity and high prevalence of CTRCD in CACS and the limitations of the few pharmaceutical treatment options based on symptoms highlights the need for alternative prevention and treatment strategies.

Exercise is a potential solution to mitigate CTRCD. Exercise decreases cardiovascular risk factors, improves cardiovascular fitness, decreases cardiac inflammation, prevents oxidative stress, and preserves cardiac structure and function at the pathophysiological level in animal models [9,16,17,18,19,20,21]. Notably, the cardiovascular-related benefits of exercise are evident from even light-intensity and voluntary exercise pre-, during, and post-cardiotoxic treatment in mice models [20]. Further, in adult breast cancer survivors, exercise is shown to decrease CTRCD as indicated by significant improvements in aerobic capacity [22,23,24,25,26,27], cardiac biomarker levels [28,29], strain [23,29], and resting heart rate [22,23,30]. Despite the plethora of evidence in preclinical animal models and adult breast cancer survivors, there is minimal research on other cancer populations, including CACS [10]. While observational studies indicate that low physical activity levels are associated with an increased incidence of cardiac dysfunction in CACS [31,32], few interventional exercise trials have been conducted. 

Before conducting future research, it is necessary to understand the extent of the current clinical studies investigating the impact of exercise on CTRCD in CACS. Therefore, the purpose of this review was to summarize the literature regarding exercise interventions aimed at mitigating CTRCD in CACS. Specifically, this review explored the breadth of exercise interventions available to manage CTRCD in at-risk CACS and how the FITT (frequency, intensity, time, and type of exercise) principle is applied to exercise interventions for CACS to manage CTRCD. The review describes the outcomes of the exercise interventions on cardiac health among CACS. 

## 2. Materials and Methods

This review was conducted per Joanna Briggs Institute (JBI) methodology for scoping reviews [33]. There was no patient or public involvement in this research’s design, conduct, reporting, or dissemination plans. A full protocol paper was submitted for publication prior to completing the review [34]. The protocol is summarized below. 

### 2.1. Search Strategy 

The first author (SJK) developed the search strategy with guidance from a JBI- trained researcher (JEL) and a JBI- trained librarian. This strategy aimed to locate published empirical studies and grey literature. The entire search strategy can be found in Table A1 (Appendix A). The search strategy aimed to identify published primary studies and reviews as well as text and opinion papers. 

### 2.2. Inclusion and Exclusion Criteria

This review included studies whose participants were CACS diagnosed at 19 years of age or younger and received anthracycline treatment and/ or chest-directed radiation therapy. Studies were required to include an exercise intervention aimed at decreasing cardiovascular disease in CACS and needed to employ a measure of cardiac surveillance at a minimum of two different time points. Studies without an exercise intervention (i.e., physical activity recall studies) were excluded. 

### 2.3. Information Sources 

The databases searched included MEDLINE, the Cumulative Index to Nursing and Allied Health Literature (CINAHL), Embase, Scopus, PsychINFO and SportDiscus. Sources of unpublished studies and grey literature were searched using the first ten pages of Google Scholar, ProQuest Dissertations, and organizational, governmental and health care association websites, including Children’s Oncology Group, PanCare, Canadian Cancer Society, American Cancer Society, National Cancer Society, Cancer Research UK, and National Health Institute (Appendix B).

### 2.4. Study Selection 

Following the search, all identified records were collected and uploaded into Covidence [35], a citation management platform and duplicates were removed. A team of four reviewers (S.J.K., J.E.L., M.A., W.J.J.) screened all titles and abstracts against the inclusion criteria. Potentially relevant papers were retrieved in total, and their full- papers were imported into Covidence. Next, the same four reviews (S.J.K., J.E.L., M.A., W.J.J.) assessed the full text of the selected citations in detail against the inclusion criteria. Reasons for exclusion of full-text articles were recorded and reported. Any disagreements were discussed between two reviewers (S.J.K. & J.E.L.). 

### 2.5. Data Extraction 

Data were extracted by a team of 3 extractors (S.J.K., J.E.L., M.A.) with at least two extractors per paper. The extraction tool was initially piloted in five studies, in which any additional aspects were discussed to retrieve from the sources. A complete extraction tool is in Appendix C. 

### 2.6. Data Synthesis and Analysis 

All data were combined to provide a complete dataset for analysis and cleaned by one reviewer (S.J.K.). The results were presented to all authors and were discussed regarding the implications.

## 3. Results

### 3.1. Study Selection

The reviewers identified 6510 records from the database search. 301, 729, 3866, 132, 160 and 611 reports were found on CINAHL, Medline, Embase, SportDiscus, PsycInfo and Scopus, respectively. All records were loaded into the review management website, Covidence. From the database search, Covidence removed 1891 duplicates, leaving 4616 articles. Two reviewers screened the title and abstract of the 4616 articles against the inclusion and exclusion criteria. Upon completion, the full text of 230 articles was assessed for eligibility, and an exclusion reason was provided for each excluded article. Reviewers identified that 64 reports focused on cardiopulmonary fitness testing, 65 did not have an exercise intervention, 28 were secondary sources, 25 did not focus on cardiotoxicity, ten did not have enough information to extract, nine were measurement validation studies, eight did not have an English full-text version, seven focused on adult cancer survivors, three focused on pharmacological treatments, three were duplicates, two assessed non-human subjects, and one did not assess cancer patients. Thus, six reports were included in the review from the database search. However, two separate papers were written based on the same study cohort and merged during the analysis [36,37]; thus, this review includes five exercise interventions.

**Figure 1:**
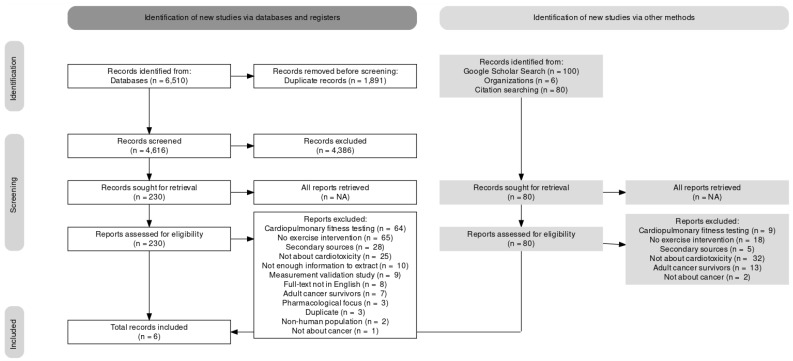
PRISMA flow diagram of included studies.

Additionally, 80 articles were identified through other search methods, including the grey literature and citation search. A single reviewer screened these articles following the above steps. The reviewer identified that nine reports focused on cardiopulmonary fitness testing, 18 did not have an exercise intervention, five were secondary sources, 32 did not focus on cardiotoxicity, 13 focused on adult cancer survivors, and two did not assess cancer patients. Thus, one record was included in the review through the other search methods. See Figure 1 for the PRISMA flow chart detailing the number of records found at each review stage.

### 3.2. Characteristics of Included Studies

Studies were published over 30 years, from 1993 to 2020. Studies were completed in the United States (n = 2), Finland (n = 1), Australia (n = 1), and Spain (n =1). Additionally, the study designs varied and included case series (n = 2), cohort (n = 2), and case-control (n = 1) reports. 

Study characteristics are included in Table 1.

### 3.3. Patient Characteristics of the Included Studies

Morales et al., 2020 was the only study that compared a control group of non-exercising CACS with an intervention group of exercising CACS and conducted a long-term follow-up [41]. The reports from Järvelä et al., 2013 and 2016 included a group of healthy controls to provide a baseline comparison with an exercising group of CACS, but the healthy controls were only assessed at baseline [36,37]. There was no control group in the other three studies [40,42,43]. 

Patient characteristics varied in the studies included in this review regarding treatment, time since diagnosis, and cancer type. In two studies, all participants were treated with anthracyclines and/ or chest-directed radiation treatment, and in the remaining three studies, most participants were treated with anthracyclines and/or radiation [40,41,43]). All study cohorts analyzed CACS, and various cancer types were included across the study cohorts. Four studies focused on survivors, and one on CACS currently undergoing treatment [41]. Finally, each study included a similar number of males and females, except the Morales et al., 2020 study, whose study sample included more males (n = 124) than females (n = 65) [41].

Study patient characteristics are summarized in Table 2. 

### 3.4. Exercise Intervention Characteristics of the Included Studies

All reviewed exercise interventions included resistance and aerobic exercise training [36,37,40,41,43], except Sharkey et al., 1993, which only included aerobic training [42]. The frequency of exercise in the reviewed studies varied from two to five sessions per week, with most studies asking participants to complete three exercise sessions per week but would allow for two sessions when necessary. For studies including a resistance training component, exercise intensity and time widely varied. However, aerobic training was generally 30 to 45 min of moderate to vigorous-intensity aerobic training, except for Long et al., 2018, which was shorter in duration [40]. Exercise interventions were based out of the participant’s home [36,37], the hospital [41], or both [42]. Long et al., 2018 did not state the setting of the exercise intervention [40]. 

Study exercise intervention characteristics are summarized in Table 3.

The studies used a variety of techniques to determine CACS’ heart health. Measurements included echocardiography to assess left ventricular ejection fraction (LVEF) [36,41,43] and tissue doppler imaging to measure mitral annulus valve velocity [36]. Other measurements included velocity vector imaging to assess strain [36], echocardiography using M-mode to assess fractional shortening [36,41], and cardiopulmonary exercise-based testing using a re-breathing technique to estimate cardiac index, defined as the cardiac output divided by body surface area [44], and stroke volume from cardiac output [42].

Three studies investigated the direct impact of the exercise intervention on LV function [36,41,43]. Of these three, only Morales et al., 2020 investigated the impacts of exercise on heart health during treatment in CACS and included a long-term follow-up of the patients [41]. The results of this study indicated that there was not a significant decline in LVEF or fractional shortening in CACS who exercised, while CACS who did not exercise saw a significant decline in LVEF (*p* < 0.001) and fractional shortening (*p* < 0.001). However, LVEF and fractional shortening decreased in CACS who exercised at the 1-year follow-up and after. Similarly, Smith et al., 2013 observed that LVEF markedly improved in all five participants upon completing an exercise intervention (median ΔLVEF = 38.2%, range: 7.6 to 56.9) [43]. In contrast, Järvelä et al., 2016 found that LVEF was not affected by the exercise intervention (*p* = 0.82) [36]. Although, other measures of LV function in this study were affected, including early diastolic mitral inflow velocity (*p* < 0.01) and early diastolic mitral annulus velocity (*p* < 0.01), indicating that the exercise intervention improved LV function. 

Sharkey et al., 1993 investigated the impact of the exercise intervention on heart health using cardiac index and stroke volume [42]. This study indicated that the exercise intervention did not significantly change cardiac or stroke volume indices. A summary of the findings related to heart health is presented in Table 4

### 3.5. Key Findings of the Included Studies Relating to Peripheral Cardiovascular Health

The studies also used various techniques to assess CACS’ peripheral cardiovascular health. Measurements included echocardiography for the left common carotid artery intima-media thickness [37], ultrasound to determine flow-mediated dilation of the left brachial artery [37,40], and a cardiopulmonary exercise test to determine peak oxygen pulse [42]. 

Three studies investigated the impact of the exercise intervention on peripheral cardiovascular health [37,40,43]. Järvelä et al. found that in survivors, the intima-media thickness significantly decreased (*p* = 0.02), and the flow-mediated dilation 40-s time point (*p* = 0.01) increased after the exercise intervention, although the specific flow-mediated dilation values were not reported [37]. Similarly, Long et al. found that in survivors, flow-mediated dilation (*p* = 0.008) significantly increased after the exercise intervention and the change in time to peak brachial diameter (*p* = 0.031) significantly decreased [40]. Smith et al. observed that peak oxygen pulse, defined as peak oxygen consumption divided by the corresponding heart rate, markedly improved in all five of their participants upon completing an exercise intervention (median Δ oxygen pulse = 25.8%, range: 6.3 to 58.6) points [43]. A summary of the findings related to peripheral heart health is presented in Table 5. 

## 4. Discussion

This is the first scoping review exploring the impact of exercise interventions on the development of CTRCD in CACS. The review identified five published studies (six reports) that met this review’s inclusion and exclusion criteria. Within these studies, exercise interventions and their impact on CTRCD varied. In brief, exercise interventions included only aerobic [42] or a combination of aerobic and resistance training [36,37,40,41,43]. Most studies (4/5 = 80%) reported positive findings suggesting that exercise may help manage CTRCD in CACS [36,37,40,41,43]. 

### 4.1. Impact of Reviewed Exercise Interventions 

The impact of exercise on CTRCD were assessed using various measurements across the five studies. All studies but Sharkey et al. [42] found that exercise significantly improved heart [36,41,43] and periphery cardiovascular health [36,40,43]. Such findings align with the current literature on healthy children. A recent interim report from the Cardiovascular Risk in Young Finns Study from 1994 to 2011 indicates that high amounts of physical activity are associated with improved left ventricular function in adulthood, as indicated by echocardiographic measurements [45]. Furthermore, Unnithan et al., 2018 found that child soccer players have significantly greater left ventricular end-diastolic volume than infrequently active controls [46].

Additionally, the findings align with the current literature on adults with cancer. Kirkham et al., 2018 found that a multi-modal exercise intervention administered during adjuvant chemotherapy treatment in early-stage breast cancer patients mitigated CTRCD, specifically exercise prevented increases in resting heart rate, hypotension, tachycardia, and impaired heart rate recovery [30]. Such findings indicate that exercise can prevent cardiac dysfunction by initiating physiological adaptations, including increased cardiac fibre contractility, leading to enhanced cardiac output and a healthier heart [47]. However, in CACS, the benefits of exercise regarding CTRCD are not entirely understood and exhibit inconsistencies. For example, Järvelä et al., 2016 indicated that exercise does not improve LVEF or fractional shortening in CACS [36], while Morales et al., 2020 indicated that exercise significantly maintains LVEF and fractional shortening [41]. 

Despite the current guidelines from the Children’s Oncology Group recommending frequent echocardiograms and cardiovascular monitoring of CACS [48], such intensive screening protocols are challenging to implement because of limited access to cardio-oncology services, infrastructure, interest, and educational opportunities [49]. Moreover, many survivors remain in primary care and do not have access to dedicated survivorship clinics or services. Furthermore, cardiac surveillance measures, such as LVEF, often do not indicate damage until significant and irreversible maladaptation occurs [15,50,51]. Thus, CACS may develop extensive CTRCD before the damage is detected. Additionally, cardiac imaging may not be sensitive enough to detect the positive effects of an exercise intervention on cardiac health in CACS. More in-depth cardiac profiling using biomarkers, such as high sensitivity troponin and natriuretic peptides, combined with cardiac imaging, as proposed by Cardinale et al., may better detect CTRCD and possibly exercise-induced cardiac adaptations [50]. 

### 4.2. Exercise Intervention Designs of Reviewed Studies

In this review, exercise intervention designs consisted of aerobic and resistance training, but the specifics of the exercise prescription varied. Most reports investigated CACS four to 30 years after receiving their diagnosis [36,37,40,42,43], while Morales et al., 2020 investigated children receiving treatment [41]. 

As many exercise guidelines for cancer survivors exist, it is worthwhile to contrast them with the studies reviewed here. The Järvelä 2013 & 2016 [36,37], and Smith et al., 2013 [43] reports aligned with the American College of Sports Medicine Guidelines suggesting that adult cancer survivors complete moderate to vigorous-intensity aerobic training for 75 to 150 min, and resistance training 2 to 3 times, per week [52]. Sharkey et al., 1993 [42] and Long et al.’s, 2018 [40] exercise intervention did not meet the American College of Sports Medicine exercise guidelines. However, Long et al.’s exercise intervention still demonstrated improved brachial artery flow-mediated dilation [40], suggesting the exercise intervention mitigated some CTRCD risk. Furthermore, the Morales et al., 2020 [41] report aligned with the pediatric oncology exercise guidelines that aerobic training should be completed 2 to 5 times per week at a moderate to vigorous intensity for 20 to 70 min and resistance training should be completed 2 to 3 times per week at a high intensity for 20 to 30 min [53]. None of the reviewed studies met the current guidelines for CACS exposed to cardiotoxic treatments. These guidelines indicate that adults should complete 2.5 h per week and children should complete one hour per day of aerobic exercise, and all CACS should also perform strength training twice per week. However, all reviewed reports demonstrated some benefits of exercise in mitigating CTRCD risk, except Sharkey et al., 1993 [42], suggesting that any exercise can mitigate CTRCD. CACS should be encouraged to exercise even if they cannot meet the guidelines and will still reap cardioprotective benefits. 

### 4.3. Limitations

A drawback of this scoping review was that three of the five [40,41,43] reviewed studies did not meet the review’s full inclusion and exclusion criteria, which required all participants to have received anthracycline and/or radiation therapy. The reviewers opted to include these three studies as the nature of the review was to map out the literature, and these studies met all other inclusion and exclusion criteria. Further, the few studies to review indicate the importance of more clinical research in this area.

Another limitation of this review was the different methods of the included studies. While most studies indicated that the exercise intervention improved cardiac outcomes, nine measures were used to assess cardiac health across the five included studies. Cardiac surveillance methods of the reviewed studies included measures such as LVEF [36,41,43], strain [36] and fractional shortening [36,41]. Additionally, the reviewed studies used a variety of measures regarding peripheral cardiovascular health, such as brachial artery intima-media thickness [37] and flow-mediated dilation [37,40]. Thus, evaluating the efficacy of the exercise interventions to prevent CTRCD was challenging as no standardized measurements were used. 

Furthermore, the reviewed studies included a wide variety of participants. The reviewed study dates occurred within a similar time frame, except Sharkey et al., 1993 [42], published nearly 30 years before Morales et al., 2020 [41]. Compared with 30 years ago, anthracyclines are used with more awareness of cardiotoxicity and better dose limitation, and advancements in radiotherapy techniques allow for decreased exposure of healthy tissues to radiation [11]. Similarly, the studies occurred in four different countries and assessed many different cancer types. As many treatment protocols are not internationally regulated and vary across cancer types, participant heterogeneity was high. Thus, comparing all studies was challenging, and these limitations should be considered when interpreting the results. 

## 5. Conclusions

This review indicates that exercise may be a viable treatment to mitigate/manage CTRCD in CACS. Further, the included studies varied widely concerning exercise intervention design, suggesting that any amount and type of exercise could help manage CTRCD. Finally, very few exercise intervention studies monitoring cardiac health have been conducted in CACS, and thus, extensive clinical research is necessary to increase the homogeneity and applicability of findings. 

Notably, the results of this review highlight the importance and benefits of exercise for CACS in preventing and managing the development of CTRCD. As the reviewed exercise interventions for CACS vary, CTRCD can be managed by various forms of physical activity and movement, and CACS should engage in exercise and become more physically active to mitigate CTRCD risk.

## Figures and Tables

**Table 1:** Characteristics of included studies.

Study Identification	Title	Country	Design	Aim	Criteria
Järvelä 2013 [37] & 2016 [36]. Methodologies as cited in [38].	Endothelial function in long-term survivors of childhood ALL: Effects of a home-based exercise program [37]; Home-based exercise training improves LV diastolic function in survivors of childhood ALL: A tissue doppler and velocity vector imaging study [36].	Finland	Case-control study	Assess the effects of a home-based exercise intervention on endothelial structure in survivors of childhood ALL [37]; Determine the effects of an exercise program on anthracycline-induced cardiotoxicity as assessed by tissue doppler imaging and velocity vector imaging in long-term childhood ALL survivors [36].	Age < 16 years at diagnosis, currently age 16–30 years, first continuous remission without hematopoietic bone marrow transplantation, diagnosed in 1986 or later, treated according to the Nordic regimen [39], and no down syndrome diagnosis.
Long 2018 [40]	Exercise training improves vascular function and secondary health measures in survivors of pediatric oncology related cerebral insult.	Australia	Cohort study	Assess the feasibility and effectiveness of a 24-week exercise intervention on cardiovascular health in childhood cancer survivors.	>5-year survivor of pediatric cancer-related cerebral insult, currently aged 15–23, not pregnant and without a current cardiovascular disease diagnosis.
Morales 2020 [41]	Inhospital exercise benefits in childhood cancer: A prospective cohort study.	Spain	Cohort study	Assess the effects of aerobic and resistance training in children with leukemia receiving neoadjuvant or intensive chemotherapy.	Currently aged 4–18 years, received a new cancer diagnosis, diagnosed, treated, and followed at the Hospital Infantil Universitario Nino Jesus, and not currently participating in any other interventional trials.
Sharkey 1993 [42]	Cardiac rehabilitation after cancer therapy in children and young adults.	United States	Case series	Assess childhood cancer survivors using exercise testing before and after a 12-week aerobic exercise program.	Received >100 mg/m^2^ of anthracyclines, post-pubertal, ≥1-year post-treatment, and no residual malignancies.
Smith 2013 [43]	Exercise training in childhood cancer survivors with subclinical cardiomyopathy who were treated with anthracyclines.	United States	Case series	Assess the effects of a 12-week exercise program on anthracycline-treated childhood cancer survivors with subclinical cardiomyopathy.	18 years of age, ≥10 years post-diagnosis of childhood cancer, treated with doxorubicin and/or daunorubicin, sedentary (<150 min of moderate-intensity physical activity per week), LVEF ≥ 40 and ≤55%, and not receiving cardiomyopathy treatment or received radiation therapy.

Abbreviations: ALL, acute lymphoblastic leukemia; LV, left ventricle; LVEF, left ventricle ejection fraction; mg/m^2^, milligrams per meter squared.

**Table 2:** Cancer participant characteristics.

Study ID	Group	Participants (Number)	Anthracyclines Dosage (mg/m^2^)	Radiation Field (Gy)	Age (Years)	Time Since Diagnosis (Years)	Cancer Type
Järvelä 2013 & 2016 [36,37]	N/A	M=10 F=11	n = 21 (Med = 240, range: 120–370)	n = 5 (unspecified dosage)	Med = 21.1 (range: 16.0–28.4)	Med = 15.9 (range: 11.3–21.4)	ALL
Long 2018 [40]	N/A	M=6 F=7	n = 4 (unspecified dosage)	n = 8 (unspecified dosage)	Med = 19 (range: 16–23)	Med = 15 (range: 7–22)	Brain = 9, ALL = 3, Other = 1
Morales 2020 [41]	Controls	M=63 F=38	n = 41 (unspecified dosage)	n = 30, (range: 1–≥50)	mean = 11 (range: 4–18)	On treatment	15 various types
	Exercise	M=61 F=27	n = 27 (unspecified dosage	n = 27 (range: 1–≥50)	mean = 11 (range: 4–17)		
Sharkey 1993 [42]	N/A	M=5 F=5	n = 10 (mean = 349 ± 69	n = 9 (range: 18–55)	mean = 19+/−3	mean = 11 (range: 4–18)	5 various types
Smith 2013 [43]	N/A	M=3 F=2	n = 4 (range: 5, 298)	n = 0	Range: 33–41	Range: 25–30	Osteosarcoma = 4, Ewing sarcoma = 1

Abbreviations: N/A, not applicable; M, male; F, female; Med, median; mg/m^2^, milligrams per meter squared; Gy, Gray; ALL, acute lymphoblastic leukemia; mean.

**Table 3:** Exercise intervention characteristics for childhood cancer survivors.

Study ID	Mode	Frequency (Sessions/Week)	Intensity	Time (min)	Type	Location	Duration (Weeks)	Instructor
Järvelä 2013 & 2016 [36,37]. Exercise protocol as cited in [38]	Resistance	3–4	3 sets, as many repetitions as possible, no rest stated.	Not stated	Eight exercises to strengthen the gluteal, lower limb, shoulders, upper limb, abdominal, and back muscles.	Home	12	Experts in sports science
	Aerobic	At least 3	Not specified	30	Participant choice (i.e., walking or jogging).			
Long 2018 [40]	Resistance	2 to 3	3 sets, 10 repetitions, 60–70% 3-RM, with 3 to 5 min of rest between exercises.	75–80	Circuit including 6 to 10 exercises targeting the chest, back, shoulders, arms, and legs.	Not stated	24	Exercise physiologist
	Aerobic	2 to 3	40–60% HRmaxwith individualized progressive increase.	10–15	Three sets of 4 consecutive sprint-rest bouts, with 3 to 5 min of rest between each set. Rowing ergometer, stationary bike, or arm ergometer.			
Morales 2020 [41]	Resistance	2 to 3	1 to 3 sets of 6–15 repetitions, 5% to 10% load increases as needed with 1 min rest between sets.	30	Shoulder, chest and leg press, side-arm rowing extension and flexion, knee extension and flexion and abdominal, lumbar and shoulder adduction.	Hospital	Med duration 22 weeks (IQR: 14, 28)	Exercise physiologist
	Aerobic	2 to 3	65–80% HRreserve with individualized progressive increase.	30–40	Ten minutes each of cycle ergometer leg pedalling, treadmill running, or arm cranking in those missing a lower limb. Ten minutes of aerobic games.			
Sharkey 1993 [42]	Aerobic	Two sessions for weeks 1–6 and 3 sessions for weeks 7–12.	60% to 80% HRmax progressive increase.	45–60	Not stated	Hospital and home	12	Not stated
Smith 2013 [43]	Resistance	3–5	1 set of 12–15 repetitions on 8 to 10 exercises.	Not stated	Not stated	Home	12	Exercise physiologist
	Aerobic	2–3	40–70%HRreserve.	20–45	Not stated			

Abbreviations: min, minutes; Min, minimum; Max, maximum; RM repetitions maximum; reps, repetitions; HR, heart rate; HRR, heart rate reserve; Med, median; IQR, interquartile range.3.5 Key findings of the included studies relating to heart health.

**Table 4:** Key cardiovascular health-related findings.

Study ID	LVEF	Valve Velocity	Strain	FS	CI	SV
Järvelä 2013 & 2016 [36,37]	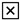	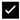 ↑	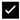 ↑	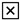	N/A	N/A
Long 2020 [40]	N/A	N/A	N/A	N/A	N/A	N/A
Morales 2020 [41]	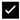 ↑	N/A	N/A	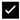 ↑	N/A	N/A
Sharkey 1993 [42]	N/A	N/A	N/A	N/A	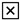	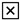
Smith 2013 [43]	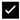 ↑	N/A	N/A	N/A	N/A	N/A

Abbreviations: 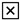, insignificant change; ↑, improvement 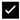, significant change; LVEF, left ventricle ejection fraction; FS, fractional shortening; CI, cardiac index; SV, stroke volume; N/A, not applicable.

**Table 5:** Key periphery cardiovascular health findings.

Study ID	IMT	FMD	Oxygen Pulse
Järvelä 2013 & 2016 [36,37]	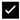 ↓	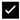 ↑	N/A
Long 2020 [40]	N/A	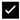 ↑	N/A
Morales 2020 [41]	N/A	N/A	N/A
Sharkey 1993 [42]	N/A	N/A	N/A
Smith 2013 [43]	N/A	N/A	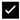 ↑

Abbreviations: 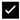, significant change IMT, intima-media thickness; FMD, flow-mediated dilation; NA, not applicable; ↑, increased; ↓, decreased.

## Appendix A

**Table A1:** Search Strategy for database search.

1	(Cancer* OR Neoplas* OR Leukemia* OR Leukaemia* OR Tumor* OR Tumour* OR Lymphoma* OR Chemotherap* OR Malignanc* OR anthracycline* OR ‘Antineoplastic Agent*’ OR Immunotherap* OR ‘Monoclonal Antibod*’ OR ‘Tyrosine Kinase Inhibitor*’ OR Radiation OR Radiology)
2	Child* OR Adolescent* OR Teen* OR ‘Young Adult*’ OR ‘Early Child*’ OR Pediatric* OR Paediatric* OR Infant* OR Toddler* OR Bab* OR Juvenile* OR ‘Pre Pubescent*’
3	1 AND 2
4	Exercise* OR ‘Resistance Training*’ OR Aerobic* OR ‘Motor Activity’ OR ‘Exercise Therap*’ OR ‘Physical Activit*’ OR Training OR ‘Physical Fitness’ OR Exertion OR Yoga OR Pilates OR ‘Dance Therap*’ OR ‘Tai Ji’ OR Qigong
5	Exp Exercise/
6	4 OR 5
7	3 AND 6
8	Myocarditis* OR ‘Heart Failure’ OR Cardiotoxic* or Cardiomyopath* OR Heart* OR ‘Radiation Injury*’
9	7 AND 8

## Appendix B

Grey Literature Check

Google Scholar

- Cancer AND Child AND Exercise AND Cardio*
- Cancer AND Pediatric AND Exercise AND Cardio*
- Cancer AND Child AND “Physical Activity” AND Cardio*

ProQuest

- cancer AND child AND exercise AND cardiotoxicity
- cancer AND pediatric AND exercise and cardiotoxicity
- cancer AND child AND ‘Physical Activity’ AND cardiotoxicity

Websites

- Canadian cancer society
- American Cancer Society
- Cancer Research UK
- National Health Institute
- American College of Sports Medicine
- Canadian Society for Exercise Physiologies
- Canadian Cardiology Society

## Appendix C

Data Extraction Instrument

- Title
- Contact
- Year
- Country
- Aim
- Design
- Start date
- End date
- Inclusion criteria
- Exclusion criteria
- Method of recruitment
- Cancer type
- Cancer stage
- Time since diagnosis
- Control group details
- Age of participants
- Chemotherapy treatment
- Radiotherapy
- Exercise program
  o Setting
  o Frequency
  o Intensity
  o Time
  o Type
  o Duration
  o Location
  o Instructor
  o Adherence
- Measure of cardiac health
- Other outcome measures
- Results
- Key findings
- Limitations
- Implications
